# Data on the volumes of water stored in the reservoirs supplying the Metropolitan Area of Sao Paulo (2003–2015)

**DOI:** 10.1016/j.dib.2018.05.049

**Published:** 2018-05-12

**Authors:** Gabriela Narcizo de Lima, Magda Adelaide Lombardo, Víctor Orlando Magaña Rueda

**Affiliations:** a Instituto de Geografía (Institute of Geography) - Universidad Nacional Autónoma de México/UNAM - Investigación Científica S.N., Ciudad Universitaria, P.C.: 04510 Mexico City, Mexico; b Instituto de Geociências e Ciências Exatas (Institute of Geosciences and Exact Sciences) – Universidade Estadual Paulista “Julio de Mesquita Filho” /UNESP, Avenida 24 A,1515, P.C.: 13506-900 Rio Claro, SP, Brazil

## Abstract

The dataset addressed in this article relates to the research article entitled “Urban water supply and the changes in the precipitation patterns in the Metropolitan Area of Sao Paulo – Brazil” (Lima et al., 2018) [1]. The dataset presents the indices of water volumes stored in the reservoirs that supply water to the Metropolitan Area of Sao Paulo (MASP). These indices were calculated based upon data provided by the Basic Sanitation Company of the State of Sao Paulo (SABESP^1^) as of the year 2003 (SABESP -Basic Sanitation Company of the State of Sao Paulo, 2017) [2]. These data include air temperature, evapotranspiration, total flow and daily withdrawal. However, due to issues related to information protection, SABESP only provides data on six of the eight water-producing systems that supply the MASP with water. Thus, the data available on the SABESP website each day were collected, processed and entered into this article calculation tables.

**Specifications Table**

**Table:** 

Subject area	*Physical Geography, Urban Planning, Climate Change*
More specific subject area	*Management of water resources*
Type of data	*Tables and graphs*
How data was acquired	• *The data regarding the volumes of water stored in the reservoirs that supply water to the Metropolitan Area of Sao Paulo were collected directly from the database of the Basic Sanitation Company of the State of Sao Paulo (SABESP) (*www2.sabesp.com.br). • *Precipitation data were provided by the Institute of Astronomy, Geophysics and Atmospheric Sciences (IAG*^2^*) of the University of Sao Paulo, which is responsible for the maintenance of the IAG Meteorological Station, belonging to the National Institute of Meteorology (INMET*^3^*)* [3].
Data format	*Data analyzed statistically and presented in the format of Excel calculation tables (.xlsx) and graphs.*
Experimental factors	*Raw data were revised in detail in order to search for missing data and/or errors of data collection.*
Experimental features	*Raw data were processed and entered into Excel calculation tables. Graphs are the result of statistical analysis of water supply data, including air temperature, evapotranspiration, total flow, daily withdrawal, and precipitation values.*
Data source location	*Metropolitan Area of Sao Paulo, Sao Paulo, Brazil*
Data accessibility	• *Raw data are partially available on the Basic Sanitation Company׳s website (www2.sabesp.com.br) and on the National Institute of Meteorology´s website (*http://www.inmet.gov.br*).* • *Processed data are available in this article.*
Related research article	*Lima, G. N.; Lombardo, M. A., & Magaña, V. O. (2018). Urban water supply and the changes in the precipitation patterns in the Metropolitan Area of Sao Paulo – Brazil. Applied Geography Journal, 94, 223 – 229.*[1]https://doi.org/10.1016/j.apgeog.2018.03.010.

**Value of the data**

• Data on the volumes of water storage in the reservoirs for MASP provide an overview of the operation of each producer system between the years of 2003 and 2015.
• These data may be used to analyze how water resources are managed in a metropolis of global importance.
• The graphs facilitate the display of data already processed, likewise, they give a clear idea of their performance during a twelve-year analysis.
• The existing data from 2003 to 2015 are already processed in this article, which means other researchers working in this area will not have to download all raw data on SABESP׳s website day by day.

## 1 Data

First, the data presented in this article refer to the gross values of the daily water volumes stored in six (6) (Cantareira, Alto Tiete, Guarapiranga, Alto Cotia, Rio Grande and Rio Claro) of eight (8) reservoirs which supply water to the MASP. It is important to mention that these data are only partially available on the SABESP website (*www2.sabesp.com.br*) (SABESP, 2017) [2]. Second, the data include precipitation totals recorded by the IAG Meteorological Station. Third, the data, monitored by the IAG and under the direction of INMET, are partially available on the site (http://www.inmet.gov.br).

One other point, raw data were downloaded and revised in detail in order to search for errors of registration and/or collection and missing data. After the revision, the data were processed statistically and entered into Excel calculation tables. The graphs are the result of statistical analysis of water supply data, including air temperature, evapotranspiration, total flow, daily withdrawal, and precipitation values. The data are presented in two categories (annual and summer values) and refer to a period of twelve (12) years (from 2003 to 2015).

## 2 Experimental design, materials and methods

The water supply at MASP is controlled daily by measurements performed in each of the producing systems that serve the region (Cantareira, Alto Tiete, Guarapiranga, Alto Cotia, Rio Grande and Rio Claro). SABESP, the public agency responsible for managing water resources, makes this gross data available on its website for public access on a daily basis.

The data presented in this article were downloaded from the SABESP site one by one, processed and entered into an Excel calculation table. Such format was chosen with the aim of facilitating display and subsequent statistical processing.

The statistical calculations aimed to estimate the means of the volumes stored in the reservoirs both seasonally and annually. Data for air temperature, evapotranspiration, total flow and daily withdrawal were also taken into consideration in this process.

The daily values of water storage in the reservoirs for MASP presented in the Excel table refer to percentages (%) in relation to the total capacity of each reservoir stored at the end of each day of measurement. To estimate this value, the total loss by evaporation and mechanical withdrawal for supply purposes are subtracted from the total volume of water available. In order to know the total amount of water available at the beginning of the process, direct measurement of the reservoir is carried out; therefore, volumes vary daily with rainfall accumulated in the days prior to this measurement.

The graphs created from these calculations were compared to the precipitation data and analyzed according to each period. Two graphs were chosen to be presented in this article as they are the most representative of the values referred to above. However, it is possible to generate graphs for daily, monthly and seasonal values from the data presented in the table.

## Footnotes

2 Acronym in Portuguese.

3 Acronym in Portuguese.
