# Supplementary material for: Data on the volumes of water stored in the reservoirs supplying the Metropolitan Area of Sao Paulo (2003–2015)
Source: Data Brief. 2018 May 12;19:409–12. doi: 10.1016/j.dib.2018.05.049 (PMC6144957; doi:10.1016/j.dib.2018.05.049)
Supplement: Supplementary file 1 — Supplementary material [file mmc1.docx]

**Data on the volumes of water stored in the reservoirs supplying the Metropolitan Area of Sao Paulo (2003 - 2015)**

Manuscript number DIB-D-18-00658

**Authors:** Gabriela Narcizo de Lima **^a^**, Magda Adelaide Lombardo **^b^**, Víctor Orlando Magaña Rueda**^a^**

**Affiliations:** ^a^ Instituto de Geografía (*Institute of Geography*) - Universidad Nacional Autónoma de México/UNAM - Investigación Científica S.N., Ciudad Universitaria, P.C.: 04510 Mexico City / Mexico.

**^b^** Instituto de Geociências e Ciências Exatas (*Institute of Geosciences and Exact Sciences*) – Universidade Estadual Paulista “Julio de Mesquita Filho” /UNESP - Avenida 24 A,1515, P.C.: 13506-900 Rio Claro – SP / Brazil.

Declarations of interest: **none**.
